# Two-Stage Revision Total Hip Arthroplasty for Periprosthetic Infections Using Antibiotic-Impregnated Cement Spacers of Various Types and Materials

**DOI:** 10.1155/2013/147248

**Published:** 2013-12-07

**Authors:** Katsufumi Uchiyama, Naonobu Takahira, Kensuke Fukushima, Mitsutoshi Moriya, Takeaki Yamamoto, Yojiro Minegishi, Rina Sakai, Moritoshi Itoman, Masashi Takaso

**Affiliations:** ^1^Department of Orthopaedic Surgery, School of Medicine, Kitasato University, 1-15-1 Kitasato, Minami-ku, Sagamihara, Kanagawa 252-0374, Japan; ^2^School of Allied Health Sciences, Kitasato University, 1-15-1 Kitasato, Minami-ku, Sagamihara, Kanagawa 252-0373, Japan; ^3^Kyushu Rosai Hospital, 1-1 Sonekitamachi, Kokuraminami-ku, Kitakyushu, Fukuoka 800-0229, Japan

## Abstract

Antibiotic-impregnated hip cement spacers of various types and materials have been used in the treatment of periprosthetic hip infections. We developed a handmade spacer by using polymethylmethacrylate (PMMA) and/or **α**-tricalcium phosphate (**α**-TCP). In this study, we retrospectively reviewed the surgical outcomes in 36 consecutive patients treated with 2-stage revision total hip arthroplasty by using our antibiotic-impregnated hip cement spacers. We aimed to analyze the infection control and reinfection rates after revision surgery. Moreover, we analyzed the possible predictors of postoperative reinfection. After exclusion of 1 patient who died immediately after the first-stage surgery, infection was controlled in 33 of the 36 hips (success rate, 91.7%). Two of these 33 hips underwent resection arthroplasty. Of the 36 hips that had been treated with the antibiotic-cement spacer, 31 hips (86.1%) were eligible for the second-stage prosthesis re-implantation. The 31 protocol hip joints of patients followed up for >6 months (mean, 48.6 months). Ten of these 31 hips (32.3%) became reinfected. No possible predictor examined differed significantly between the reinfection-positive and reinfection-negative groups. However, spacers consisting of PMMA cement alone were associated with the highest risk of reinfection. Therefore, **α**-TCP-containing antibiotic-impregnated hip cement spacers might decrease the reinfection rate in patients undergoing re-implantation.

## 1. Introduction

Periprosthetic infection of the hip is the most serious complication after total hip arthroplasty (THA) and femoral head prosthesis (FHP) replacement. It imposes physical and mental stress and an economic burden on affected patients [1]. Moreover, postoperative infection can damage the trust-based patient-physician relationship. It is therefore most important to prevent postsurgical infection or, if infection has already occurred, to treat it appropriately. In the present study, we treated late stage (≥3 months postoperatively) or early stage (<3 months postoperatively) post-THA infection characterized by repeated recrudescence despite debridement without implant removal. The first stage, we controlled using an antibiotic-impregnated cement spacer with implant removal for infection. In the second stage, we used bone allografts to restore the bone defects in cases of implant loosening and massive bone defects resulting osteolysis of infection and repeated debridement [2, 3].

Although there are various options for treatment of post-THA infection, a 2-stage protocol with insertion of a type of antibiotic spacer has been widely reported [2, 4–8]. In this study, we aimed to analyze the rates of infection control and reinfection after revision surgery for treatment of periprosthetic infections of the hip at our institution by using antibiotic-impregnated cement spacers of various types and materials. Moreover, we aimed to analyze the prognostic factors that might have influenced the development of postoperative reinfection in the patients in this series.

## 2. Materials and Methods

The study was approved by our institutional review board. From January 2000 to June 2012, we performed 2-stage revision THA, including FHP replacement, by using an antibiotic-impregnated cement spacer on 37 hips of 36 patients with infected THA. The patients comprised 19 men and 17 women (including both hips of 1 woman) with a mean age of 62.4 years (range, 27–90 years) at the time of the first-stage surgery, and who were followed up for a mean of 48.6 months (range, 6–127 months). The underlying diseases included osteoarthritis in 16 hips, femoral neck fracture in 9 hips, idiopathic osteonecrosis of the femoral head in 5 hips, rheumatoid arthritis in 2 hips, acetabular fracture in 2 hips, septic arthritis in 2 hips, and ankylosis in 1 hip. Twenty-two and 13 hips developed infection after primary THA (FHP replacement) and revision surgery for failed THA (FHP replacement), respectively. One hip developed infection after repeated revision surgery and another after resection of the products of heterotopic ossification after THA.

**Figure 1:**
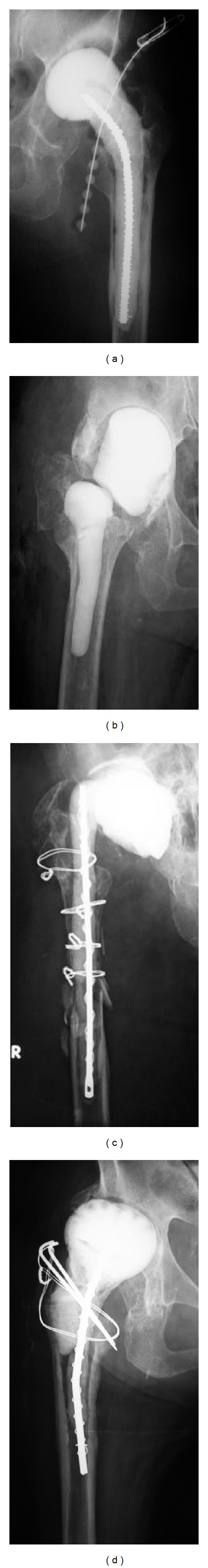
(a) The conventional polymethylmethacrylate (PMMA) cement spacer. (b) The *α*-tricalcium phosphate (TCP) spacer. (c) The separately prepared PMMA cement spacer (femoral side) and *α*-TCP spacer (acetabular side). (d) The new type of antibiotic-impregnated spacer.

Various materials have been used for spacers throughout the years. A conventional polymethylmethacrylate (PMMA) cement spacer was used in 9 hips (Figure 1(a)). An *α*-tricalcium phosphate (*α*-TCP) spacer prepared separately for the femoral and acetabular sides was used in 8 hips (Figure 1(b)). In 6 hips, the same method was used to prepare, separately, a PMMA cement spacer (femoral side) and an *α*-TCP spacer (acetabular side; Figure 1(c)). Finally, a newly devised spacer with an *α*-TCP core was used in 14 hips (Figure 1(d)). In addition, all implants were removed during the first surgical stage in 30 hips, whereas a spacer was prepared only for the acetabular side, preserving the stem, in 5 hips (a PMMA spacer in 4 hips and an *α*-TCP spacer in 1 hip). Bone defect reconstruction by using bone allografts was performed in 19 hips (61.3%) : 5 (16.1%) on the femoral side, 4 (12.9%) on the acetabular side, and 10 (32.3%) on both sides.  Table 1 presents the details of the causative organisms of the infections present in the 37 hips during the first reconstruction stage.

One patient was excluded from this study because of death caused by hypovolemic shock on day 3 after the first surgical stage. The remaining 35 patients (36 hips) were included in a survey of the rates of infection control, performance of the second stage of the revision surgery, and reinfection after the second-stage revision surgery. In addition, the patients were divided into 2 groups according to the presence or absence of reinfection at the time of the final follow-up or earlier for statistical comparison to identify the factors likely to be involved in reinfection, including the frequency of previous surgery, type of spacer used in the first surgical stage, causative bacterium, and use of a bone allograft. The Mann-Whitney *U* test and Fisher's exact test were used for statistical analysis. We adopted a significance level of *P* = 0.05.

## 3. Surgical Technique

### 3.1. The First Surgical Stage

The first stage of the surgical procedure (infection control) involved the following steps.

*(1) Prosthesis Removal, Debridement, Cleaning, and Creation of an Antibiotic-Impregnated PMMA Cement Spacer by Using α* *-TCP (Biopex, Mitsubishi Materials, Tokyo, Japan)*. All the surgeries were performed with the patient in the lateral position. The approach was preferably made via the previous surgical scar. However, when no old surgical scar was available, a new skin incision was made, with a Gibson skin incision being the most frequently used. The transtrochanteric approach was frequently used to secure a sufficient operative field. In cases of fistula, gentian violet was injected via the fistula to mark the surgical site, the fistula was then resected. Joint fluid samples were collected for bacterial culture. Synovial membrane and periarticular tissue samples were collected for bacterial culture and pathological examination. In cases of a stable stem or difficulty in removing the bone cement, extended trochanteric osteotomy with preservation of the attachments of the gluteus medius and vastus lateralis muscles onto the femur was performed [9]. Contaminated tissues on the acetabulum, around the femoral neck, and in the femoral marrow cavity were thoroughly curetted and sampled for bacterial culture and pathological examination. Granulation tissue that appeared on visual inspection to be caused by infection was curetted completely, whereas bone, except for free sequestra, was preserved as much as possible. After curettage, the lesion was washed with a large volume (more than 10 L) of saline solution by using pulsed irrigation. The spacer was prepared with reference to the shape of the hip prosthesis on a preoperative anteroposterior radiograph. The spacer was prepared by another team either in parallel with the first surgical stage or a day earlier in the same operating room (in the latter case, it was then wrapped in a sterile sheet and drape and refrigerated). After the washing, the gloves, surgical gowns, and surgical equipment used were exchanged for freshly sterilized replacements. The drape used in the operative field was also replaced.

*(2) Creation of Handmade Antibiotic-Impregnated PMMA Cement and α* *-TCP Spacers*. Gentamicin (GM) was the antibiotic of choice because it withstands the high temperature generated by cement polymerization, has a broad spectrum, does not lose activity over time, and elutes efficiently from the cement. Because GM powder was difficult to obtain in Japan, liquid GM equivalent to 1200 mg of GM (60 mg/1.5 mL × 20 ampules) was mixed with 40 g of cement, placed in a sterile pack, and dried with hot air before use. In cases of infection caused by methicillin-resistant *Staphylococcus aureus* (MRSA), the spacer was prepared by using 2.0 g of vancomycin (VCM) powder (0.5 g × 4 vials) and 40 g of a cement with a lower polymerization temperature (Cemex RX; Exactech, Gainesville, FL, USA) [10]. To prevent breakage, the spacer was reinforced with an Ender nail wrapped with a soft steel wire. When the causative bacterium was unidentifiable, 2.0 g VCM and 1200 mg GM were mixed with 40 g cement.

Since March 2005, *α*-TCP has been preferred because it was reported to generate no heat during polymerization and to allow the gradual release of the antibiotic embedded in the spacer [11]. Because FHP-type spacers composed of only *α*-TCP, which has low strength and frequently collapsed, antibiotic-impregnated *α*-TCP spacers were developed for separate placement on the femoral and acetabular sides. However, this precluded correction of leg length discrepancy during the waiting period. For this reason, new spacers using a combination of PMMA cement and *α*-TCP were developed and have been used since February 2008.

**Figure 2:**
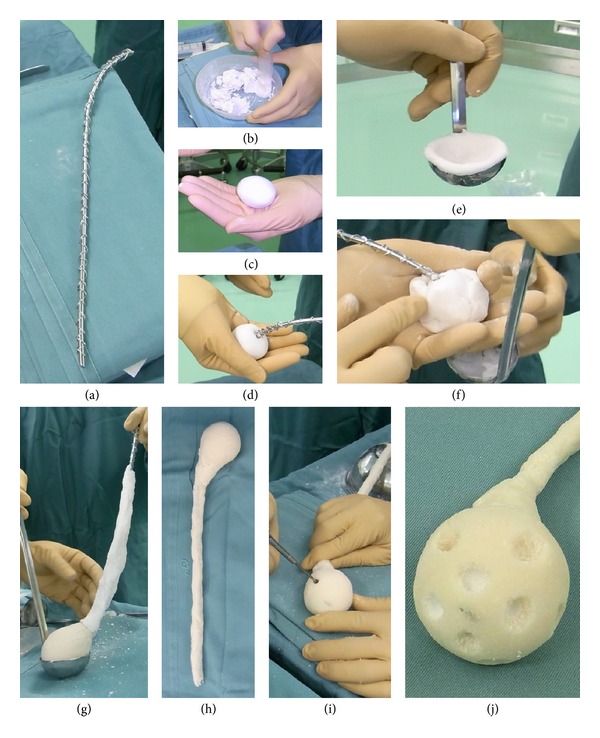
Steps in the making of the new type of handmade antibiotic-impregnated spacer.

*(3) Creation of a New Type of Handmade Antibiotic-Impregnated Spacer*. The new type of spacer was prepared similarly to the spacer made of a combination of PMMA cement and *α*-TCP, by winding a soft steel wire around an Ender nail to prevent the nail from breaking, which makes it difficult to remove the distal spacer (Figure 2(a)). The amounts of antibiotics used were 0.5 g of VCM and 60 mg (1.5 mL) of GM to 12 g of *α*-TCP powder (Figure 2(b)). The core part of the femoral head was formed from 48 g of *α*-TCP containing 2 g of VCM and 240 mg of GM (Figure 2(c)) and placed at the tip of an Ender nail (Figure 2(d)). The part of femoral head was prepared by wrapping PMMA bone cement containing 2 g of VCM with *α*-TCP and shaping it by using an appropriately sized ladle (Figures 2(e) and 2(f)). The stem needed to be prepared carefully with PMMA cement so that it did not become too thick (Figure 2(g)), although the neck part should be somewhat thick to prevent fractures (Figure 2(h)). After completion of the spacer, a pore reaching the *α*-TCP core of the femoral head was made by using an surgical airdrill to create a channel for efficient gradual release of the antibiotics (Figures 2(i) and 2(j)).

To improve the results of spacer placement, advance trial reposition was performed to check whether the femoral head would fit the acetabulum. An excessively large femoral head of the spacer can restrict hip mobility, as well as hip repositioning, postoperatively. To fill the dead space and enhance gradual antibiotic release, antibiotic-impregnated *α*-TCP was added to the neck part after placement and repositioning of the spacer. The excised portions of the greater trochanter and femoral stem were temporarily fixed by performing tension-band wiring and wiring, respectively, until the next surgery.

*(4) Systemic Administration of Antibiotics after the First Surgical Stage*. Similarly, as for the conventional surgical technique, a cephem antibiotic was administered systemically for approximately 3 days, including the day of surgery, because the local concentration of antibiotic gradually released from the spacer was sufficient after that time. Since February 2008, antibiotics effective against the causative organisms of infection were chosen and administered until the patient's C-reactive protein (CRP) level returned to within normal limits unless complications such as persistent infection by the causative organism occurred, in which case the antibiotic susceptibility of the bacteria was examined. In addition, if the causative organism of infection was MRSA, we determined the appropriate antibiotic (including polypharmacy) and the duration of systemic administration by discussion with the infectious control team of our institute. When the infection was effectively controlled, the CRP level was normalized after approximately 3 weeks. When the CRP level remained normal, the second surgical stage was planned after a waiting period of 6–8 weeks. The mean waiting period in the present study was 55 days (range, 16–215 days).

### 3.2. The Second Surgical Stage (Revision Surgery)

In the second surgical stage, the patient was maintained in the lateral position, as in the first surgical stage. After the spacer was removed, the synovial membrane of the pseudosynovial cavity formed around the spacer was curetted and sampled for bacterial culture; a joint fluid sample was also obtained for bacterial culture. Infection control was evaluated based on the presence or absence of bacteria by performing immediate pathological examination of a Gram-stained smear and a polymorphonuclear leukocyte count [12]. The bone defect was reconstructed, with the use of a bone allograft if that had been decided during the preoperative planning, and a hip prosthesis was placed.

## 4. Results

Excluding 1 patient who died immediately after completion of the first surgical stage, the infection was controlled in 33 of the 36 hips, for a success rate of 91.7%. One hip (2.8%) underwent redebridement and resection arthroplasty because of failure of infection control. Two of the 5 hips in which the stem was preserved underwent reremoval of the stem followed by debridement and placement of another antibiotic-impregnated cement spacer to control the infection. The second-stage revision surgery could then be performed because the infection had been effectively controlled. Two of 33 hips underwent resection arthroplasty. Of the 36 hips that had been treated with the antibiotic-cement spacer, 31 hips (86.1%) were eligible for the second-stage prosthesis re-implantation.

Ten (32.3%) of the 31 hips became reinfected after second-stage surgery. One patient underwent revision surgery of the acetabular side cup and replacement of the proximal part of the stem to elongate the neck after experiencing repeated dislocation after the second-stage revision surgery. However, reinfection by another bacterium occurred after the surgery, so second-stage revision surgery with a bone allograft was performed again with a good result. One patient underwent second-stage revision surgery with a bone allograft but experienced recrudescence of *E. coli* similar to the causative organism of the initial infection in the early postoperative period. The infection was eventually controlled by replacing the bone allograft with an antibiotic-impregnated *α*-TCP spacer. One patient developed reinfection by a bacterium different from the causative organism 4 years after the second-stage revision surgery. The patient underwent repeat second-stage revision surgery with a bone allograft, this time with good results. Another patient underwent rereplacement of only the cup during the first stage of the revision surgery because of recrudescence of the same bacterium that caused the initial infection. Two patients underwent repeat second-stage revision surgery because of recrudescence of the same bacterium that caused the infection after the initial second-stage revision surgery. Four patients required additional surgery because of MRSA infection after the second-stage revision surgery.

Comparison between patients with and without reinfection produced the following results. The mean number of previous surgeries was 3.2 (range, 1–7 times) in the groups with reinfection versus 2.8 (range, 1–6) in the group without reinfection. MRSA was the causative bacterium in 4 (40.0%) and 6 hips (28.6%) in the groups with and without reinfection, respectively. Of the 31 hips that underwent the second-stage revision surgery, 5 (50.0%) and 14 (66.7%) in the groups with and without reinfection, respectively, were repaired with bone allografts. A PMMA spacer was used in 4 hips each in the groups with (40.0%) and without (19.0%) reinfection. None of these possible predictors of reinfection differed significantly between the patients in the 2 groups (Table 2).

## 5. Discussion

There are several published reports on the treatment of periprosthetic infections after THA and FHP replacement. Other reported treatment options include 2-stage revision THA [4, 10], 1-stage replacement [13, 14], long-term antibiotic suppression [15], resection arthroplasty, arthrodesis [16], amputation, irrigation, and debridement with liner replacement. Antibiotic-impregnated cement beads have been reported to be effective for preventing infection after 2-stage revision [17]. However, we believe that 2-stage revision surgery comprising debridement, implant resection, implantation of an antibiotic-impregnated cement hip spacer, and delayed re-implantation is the most effective treatment for periprosthetic infections [4, 10] because it allows the maintenance of the patient's leg length and hip function as well as good infection control. In cases of mild periprosthetic infection of the hip, it is difficult to decide whether to remove the entire implant. However, it can be difficult to control periprosthetic infection while preserving the implant. In the present study, periprosthetic infection was not controlled in 2 of the 5 hips in which the stem was preserved, ultimately requiring stem removal followed by redebridement and spacer replacement. Therefore, we consider it difficult to control infection while preserving the prosthesis.

We also previously reported our institution's experience with a 2-stage re-implantation protocol. Takahira et al. [10] reported an infection control rate of 89% with the 2-stage protocol. Hsieh et al. [18] reported an infection control rate of 95.3% by using an antibiotic-impregnated hip cement spacer and beads. In contrast, Fehring et al. [19] reported a failure rate of 63% (54 of 86 patients) for treatment of periprosthetic infection by using irrigation and debridement alone. In addition, we do not currently apply continuous washing because doing so would require patients to undergo bed rest, complicate infection control, and result in lower-limb shortening. The use of an antibiotic-impregnated cement spacer is reported to produce better outcomes than irrigation. Therefore, we consider the spacer, which allows gradual local release of high concentrations of antibiotics, to be highly effective. The present results show control of the infection by using the spacer in 33 (91.6%) of the 36 hips. In addition, the second-stage revision surgery could be performed in 31 (86.1%) of the 36 hips, which is similar to the frequency previously reported [10]. In the treatment of infection, it is important to consider the systemic condition of the patient and to determine during the first-stage revision surgery whether it is necessary and advisable to remove the entire prosthesis and/or perform second-stage reconstruction.

Calcium phosphate cement (CPC) has been used for bone replacement and augmentation because of its good biocompatibility and osteoconductivity. Sasaki et al. [11] reported that CPC has the advantage of not heating up during cement polymerization. In addition, it allows the maintenance of high antibiotic concentrations within an infected lesion. The authors demonstrated that VCM-impregnated CPC was able to maintain a higher concentration of VCM in focal areas for 2 weeks than was in bone cement and indicated that VCM-impregnated CPC may be more effective than bone cement for treatment of osteomyelitis or prosthesis infections. We also used CPC for our hip spacers. However, we prepared and placed the acetabular and femoral sides of the spacer separately because of the insufficient strength of this material. Nevertheless, we observed unstable hips during the waiting period before the second-stage revision surgery and leg shortening due to insufficient allowance to maintain leg length; we also experienced difficulty removing scattered fragments of fractured CPC during the second-stage revision surgery. For this reason, we recently developed a new type of spacer with a CPC core, which we found to be highly effective for the gradual release of antibiotics and to have strength comparable to that of PMMA cement. In a future study, we will report the therapeutic performance of our new spacer against infectious diseases.

The use of bone allografts to restore bone stock in a previously infected environment is controversial. One of the main concerns of using a bone allograft to treat massive bone loss in revision hip arthroplasty for treatment of infection is the theoretically increased risk for reinfection. Conversely, the use of a bone allograft in second-stage revision surgery for treatment of infection has frequently been reported to produce good results [5, 18, 20, 21]. The present results indicate that the reinfection rate was lower in the patients in whom a bone allograft was used for bone defect in the second-stage revision surgery for treatment of infection than in the patients in whom a bone allograft was not used, although there was no clearly significant difference. Therefore, we do not consider the use of a bone allograft to be a risk factor for reinfection, and this hip reconstruction technique should be implemented actively in the future.

The rate of reinfection after the second-stage revision surgery in the present study, which included recrudescence of infection and reinfection by bacteria different from the initial causative organisms of infection, was 32.3% (10/31 hips), an inferior performance relative to previous reports [22–24]. The causative factors for infection include systemic and local factors. As improving and maintaining the patient's systemic status seems to be necessary for the prevention of reinfection, we consider it important even for orthopedic surgeons to understand the patient's systemic condition (e.g., status of blood glucose control, amount of steroid medication used, presence or absence of urinary tract infection, smoking, nutritional status, and dental health).

Massive hemorrhage and prolonged surgery are expected during hip reconstruction after infection control and impose significant stress on patients, and Berend et al. [4] reported that the mortality rates associated with the treatment of infected THA are substantial. The authors performed a 2-stage protocol in 202 patients (205 hips) with infected primary or revision THA. Fourteen patients (7%; 14 hips) died before re-implantation, and the 90-day mortality rate after first-stage debridement was 4% (8 patients). Of the 186 patients (189 hips) who underwent re-implantation, 157 (83%) achieved infection control. When all of the patients who underwent the first-stage revision surgery were included, the rate of survival and infection control after 2-stage re-implantation was 76%. Berend et al. [4] concluded that successful 2-stage treatment should include not only effective control of infection but also successful second-stage re-implantation. Infection control is not achieved if death occurs before the second-stage operation; therefore, deaths should be excluded when determining the success rate of infection control. We also experienced one case in which 1 patient died of cardiac hypofunction due to hypovolemic shock on day 3 after the first-stage surgery. This case illustrates the importance of fully understanding the patient's systemic condition and likelihood of tolerating surgery and of providing sufficient explanation to the patient and his or her family before obtaining consent to undergo surgery. Moreover, we consider it necessary in the future to provide mental health care to patients who develop infection in order to mitigate their uneasiness about unforeseeable treatment outcomes and the necessity of long-term hospitalization.

## 6. Conclusion

The examined possible predictors of postoperative reinfection did not differ significantly between the reinfection-positive and reinfection-negative groups; however, the use of only a PMMA cement spacer was associated with the highest risk of reinfection. In the treatment of infection, it is important to determine whether or not the entire prosthesis needs to be removed during the first-stage revision surgery.

## Figures and Tables

**Table 1:** Details of the causative organisms of infection in the 37 hips during the first-stage revision surgery.

Organism (*n* = 37)	No. of patients, *n* (%)
*Staphylococcus epidermidis *	10 (27.0)
Methicillin-resistant *Staphylococcus aureus* (MRSA)	9 (24.3)
*Staphylococcus species *	5 (13.5)
Methicillin-sensitive *Staphylococcus aureus* (MSSA)	2 (5.4)
*Escherichia coli *	2 (5.4)
Group B *streptococcus *	2 (5.4)
*Klebsiella *	1 (2.7)
Polymicrobial organisms	1 (2.7)
Unknown	5 (13.5)

**Table 2:** Comparison of the possible prognostic factors of re-infection between the re-infection-positive and re-infection-negative groups after the second-stage revision surgery in 31 hips.

	Re-infection negative (21 hips)	Re-infection positive (10 hips)	*P* value
No. of previous operations	2.8 ± 1.5	3.2 ± 1.7	0.467
Use of an allograft	14/21 (66.7%)	5/10 (50.0%)	0.308
Infection by MRSA	6/21 (28.6%)	4/10 (40.0%)	0.405
PMMA cement spacer	4/21 (19.0%)	4/10 (40.0%)	0.208

MRSA: Methicillin-resistant *Staphylococcus aureus.*

PMMA: Polymethylmethacrylate.
